# Decision-making trades off learned and perceived information

**DOI:** 10.21203/rs.3.rs-7972005/v1

**Published:** 2025-10-29

**Authors:** Tal Nahari, Boaz Rozenberg, Yoni Pertzov, Eran Eldar

**Affiliations:** 1 – Cognitive and Brain sciences, Hebrew University of Jerusalem; 2 – Affective Brain Lab, Department of Experimental Psychology, University College London, London, UK; 3 - Max Planck UCL Centre for Computational Psychiatry and Ageing Research, University College London, London, UK; 4 – Psychology Department, Hebrew University of Jerusalem

**Keywords:** decision making, eye tracking, value learning, information gathering

## Abstract

A fundamental question in cognitive science is how information from internal memory is combined with external sensory input when making decisions. We hypothesized that previously learned and currently perceived information trade off against each other, such that extracting information from one source reduces the gathering and usage of information from the other. To test this hypothesis, we designed a two-armed bandit task where each arm is composed of both learned and perceived elements. We monitored participants’ gathering of perceptual information using eye tracking. Participants’ choices and gaze deployment showed a trade-off between the impact of learned and perceived information. The more a participant utilized internally stored learned information, the less they gathered perceptual information, and vice versa. Modeling participants’ information gathering indicated that the trade-off results from the faster gathering of learned information, which, when used, makes it less valuable to further invest effort in gathering additional perceptual information. Preliminary findings also suggested that an individual’s tendency to primarily rely on one source of information is a stable individual trait. These findings reveal how humans balance between learning and perception in forming decisions.

The many decisions we make every day may rely on information from the environment that we perceive using our senses (e.g., deciding whether to take an umbrella by looking outside to see whether it is cloudy), and on previously learned information that we gather from within our minds (e.g., recollecting whether it normally rains at this time of the year^1^). Prior research extensively examined how people use either source of information in separate, yet it remains unclear how people integrate information from both sources.

Answering this question is difficult not only due to a lack of empirical data but also because research on perceptual and learning-based decisions has largely relied on diverging modeling frameworks^2^. Perceptual decision-making has most prominently given rise to models of gradual evidence accumulation where the proclivity to take one or another decision evolves over time during the decision process^3–5^. Conversely, learning-based decisions have mostly been studied through the lens of reinforcement learning^6–10^, which explains how preferences change from decision to decision, but not during the decision process. That said, substantial research has shown how learning-based decisions may also form gradually, through the accumulation of internally sampled information^11–15^. Perceptual and leaning-based decision-making may therefore share a common algorithmic form, and potentially require overlapping cognitive resources.

One key difference, however, is that visual stimuli are typically sampled progressively via eye movements^16^, whereas the gathering of learned information is limited only by the speed of neurons. If these two sources of information are gathered at comparable speeds, the trade-off between them can be expected to be symmetric, such that gathering more information from one source means gathering less of the other. However, if learned information is gathered faster, as it does not require operating ocular muscles, learning could enable faster decisions and reduce the residual value of investing additional time in gathering perceptual information. Thus, here we ask whether there is a trade-off between the use of learned and perceived information in making decisions, and if there is, whether it is symmetric or directional.

To answer this question, we designed a novel decision-making task that required jointly utilizing learned and perceived information to maximize reward (preregistered at https://osf.io/78vqu). Importantly, sensory information was collected by participants by shifting their gaze, as consistent with how such information is gathered in the wild. This also enabled us to precisely monitor the gathering process via eye tracking. As anticipated, we found that the two sources of information traded off against each other. However, the tradeoff was directional — learned information influenced the amount of perceptual information that was gathered to reach a decision. Joint modeling of choices and eye movements revealed the mechanism at play - the more informative the learned information, the less participants gathered perceptual information. Preliminary evidence also indicated that the tendency to rely on either learned or perceived information is a stable individual trait.

## Results

88 individuals completed the task, out of which 72 provided full eye tracking data. In each trial, participants chose between two stimuli, each composed of two elements (Figure 2): a colored circle and a surrounding ring of 12 Landolt Cs, each facing either upwards or downwards. The colored circle was associated with a fixed probability of reward that could only be learned through trial and error. By contrast, with regards to the surrounding Landolt C’s, participants were informed that the probability of reward scales linearly with the number of Cs that face upwards. Thus, whereas the colored circle could be instantaneously identified using peripheral vision but required consulting learned information to estimate associated reward probability, the Landolt Cs’ required protracted perceptual processing to identify, but their associated reward probabilities were precisely known. Importantly, participants were made aware that the color of the circle and the number of Cs facing upwards bared equal weights in determining reward probability associated with each stimulus, and thus both learned and perceived information were equally important for maximizing reward.

### Participants jointly used learned and perceived information

**Figure 1.**
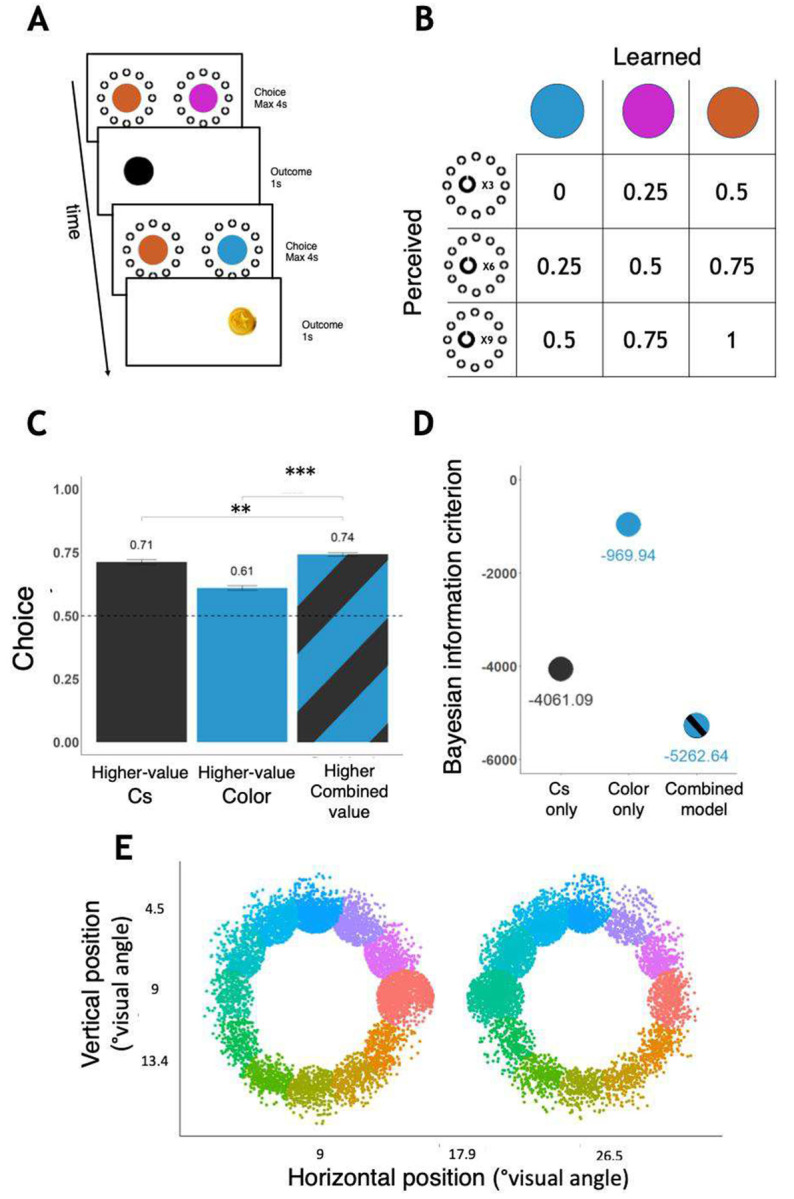
Participants used both color and Cs to make decisions. A. Timeline of two trials. In each trial, participants chose either the left or right stimulus, and subsequently received or did not receive a reward (gold coin). B. Probability of reward as a function of color and number of upward facing Landolt Cs. The probabilities associated with the colors could be learned by trial and error, whereas those associated with the Landolt Cs were instructed. In the first two blocks there were also trials where stimuli were composed of Cs only or colors only (see supplementary materials). C. Participants tended to choose stimuli with higher combined reward probability, more so than they tended to choose stimuli with more upward-facing Cs or higher color reward probability. Error bars indicate s.e.m. **: p<.01, ***: p<.001. D. Bayesian information criteria for three regression models explaining participants’ choices. The model utilizing both Landolt Cs and colors fitted choices better than models using either source of information alone. E. Classification of participants’ fixations based on the nearest Landholt C it landed next to.

We first validated that participants utilized both perceived and learned information when making a decision. To test that, we compared participants’ tendency to choose the stimulus with the higher total reward probability, accounting for both Cs and colors, to their tendency to simply choose the stimulus with the more upward-facing $\text{Cs}\left(t_{87}=3.19,p=0.002\right)$, and to their tendency to choose the stimulus with the more rewarding color $\left(t_{87}=15.47,p<.001\right)$. The results confirmed that participants accounted for both sources of information in making their choices (Figure 1C).

In addition, to determine whether the use of both Cs and color explained participants’ choices better than either the Cs or the color separately, we compared three regression analyses explaining participants’ choices by: (1) the difference between the two available stimuli in the number of upward Cs; (2) the difference between the stimuli in the reward probability associated with the color; and (3) both. A $\chi^{2}$ test showed that the Cs and color together explained choices better than either one separately ($\chi^{2}(4)$ of combined vs. Cs: 1241.2, p<.001; $\chi^{2}(4)$ of combined vs. color: 4332.4, p<.001). Additionally, both Cs and color predictors were significant $\left(\beta_{\text{Cs}}=1.54,z=55.5,p<.001;\beta_{\text{color}}=0.73,z=28.3,p<.001\right)$. Lastly, the same conclusion was also obtained by comparing the regression models using the Bayesian information criterion (BIC; Figure 1D).

### Trade-off between learned and perceived information within participants

Having verified that participants used both learned and perceived information to make their choices, we next tested whether a trade-off existed between them within participants, such that when a participant used more learned information, they gathered less perceived information, and vice versa. For this purpose, we leveraged the monitoring of participants’ perceptual information gathering via eye-tracking. We first confirmed that the Cs participants fixated on, as identified by eye tracking, explained participants choices better than accounting for all the Cs that appeared on the screen (Table S2). Then, we regressed participants choices on: (i) the difference between the two choice options in the number of upward minus downward Cs participants fixated on ($\Delta {\text{Cs}}_{\text{observed}}$); (ii) the difference between the choice options in the reward probability associated with the stimuli colors (color); and (iii) the trade-off between using the colors and looking at Cs – an interaction between the color predictor and the total number of all Cs the participant fixated on during the trial, denoted color ×ΣCs. We did not include here the total number of Cs participants fixated on as a predictor outside of the interaction because we did not expect it to have a direct effect on choice, as confirmed in a supplementary analysis (see Figure S2).

**Figure 2.**
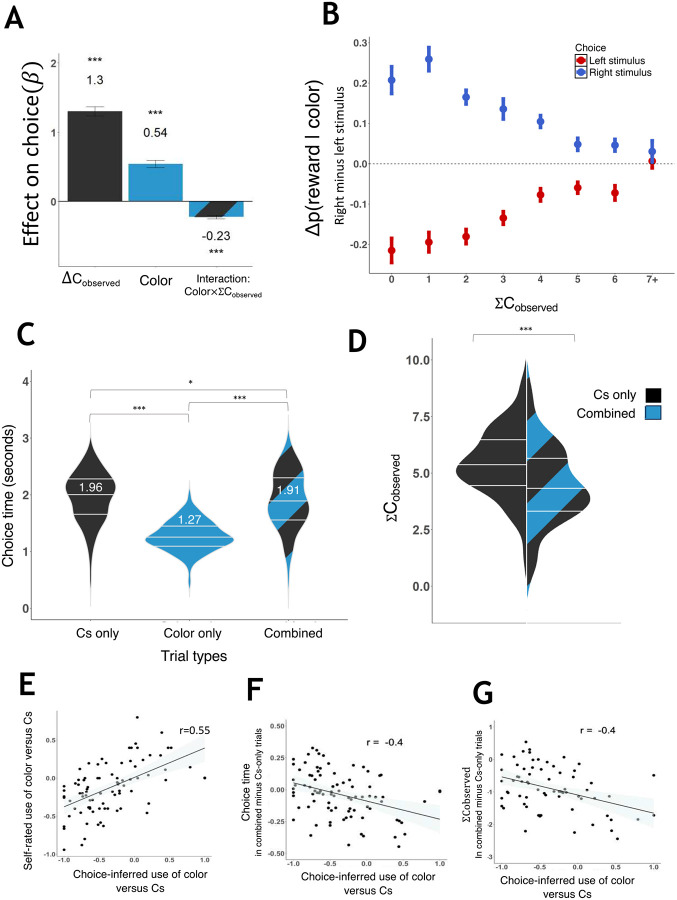
Trade-off between color use and looking at Cs. *: p<.05. ***: p<.001. A. Regression coefficients of a mixed logistic model explaining choices based on the Cs participants fixated on (upward facing minus downward facing, right minus left stimulus), the reward probability associated with the colors (right minus left stimulus), and an interaction between the color predictor and the total number of Cs participants fixated on. Error bars indicate standard errors across participants. ***: p<.001. B. The difference between the reward probabilities associated with colors of the right and left stimuli as a function of which stimulus was chosen and the total number of Cs participants fixated on. When participants made fewer fixations, they exhibited a stronger tendency to choose the more rewarding color. Lines represent standard error across participants. **C**. Mean choice time as a function of trial type. Lines depicts the three quartiles (0.25, 0.50, 0.75) of each distribution, and numbers denote the mean. D. Mean total number of Cs fixated on per trial as a function of trial type. Fewer Cs were fixated on in the trials combining both colors and Cs, relative to Cs only trials. Lines depict the three quartiles (0.25, 0.50, 0.75) of each distribution. E. The use of color relative to Cs as inferred from participants’ choices correlated with participants’ own ratings at the end of the experiment as to which source of information they relied more on. The relative use of color and Cs was calculated based on choices as $\frac{\beta_{\text{color}}-\beta_{c}}{\left(\left|\beta_{c}\right|+|\beta_{\text{color}}\right)}$ using the regression coefficients derived from the combined color and Cs regression in Figure 1C. F. Choice time in combined minus Cs only trials as a function of individual differences in the use of color relative to Cs in choosing between stimuli. G. The number of Cs participants fixated on in combined minus Cs only trials as a function of individual differences in the use of color relative to Cs in choosing between stimuli.

We found that both Cs and colors influences choice, and most importantly, the interaction was negative, meaning that the more participants used learned information the less they gathered perceived information (Figure 2A, Figure S2).

Next, we ruled out that this interaction was due to the higher strength of evidence that a higher number of Cs could offer in favor one or the other stimulus. According to this alternative interpretation, the tradeoff results because participants use the colors less when the strength of perceptual evidence is higher. We tested this interpretation by adding to the previous regression another interaction - between the color predictor and the strength of perceptual evidence, that is, the absolute difference between stimuli in the upward- minus downward-facing Cs that participants fixated on (denoted: $\left|\Delta {\text{Cs}}_{\text{observed}}\right|\times \text{color}$). In this expanded regression, we only found a significant effect for the interaction with the total number of Cs participants observed and not with the strength of the evidence these Cs offered ($\beta_{\Delta {\text{Cs}}_{\text{observed}}}=1.31,z=19.7,p<.001,\beta_{\text{color}}=1.56,z=0.34,p<.001$, $\beta_{\text{color}\times \Sigma \text{Cs}}=-0.22,z=-7.94,p<.001,\beta_{\left|\Delta Cs_{\text{observed}}\right|\text{Xcolor}}=-0.03,z=-0.69,p=.48$). Thus, the use of learned information for making choices traded off specifically against the amount of perceptual information gathered.

### Learned information reduces sampling of perceptual information

Next, we asked why a trade-off exists between the use of perceived and learned information. A natural suggestion is that the trade-off is caused by the fact that gathering each of the two kinds of information requires time and uses overlapping cognitive resources, such that it is slower to gather both of them simultaneously. If this is the case, we may expect the time it takes to gather both kinds of information to be longer than the time it takes to gather each of them separately. However, comparing choice times on trials where stimuli consisted of either Cs alone, color alone, or both showed that the addition of color to the Cs made participants reach decisions faster, not slower (combined vs. Cs only: $t_{87}=2.21,p=0.03$; Figure 2C), despite the fact that to choose between combined stimuli optimally, both Cs and color needed to be consulted, and participants had plenty of time left in the trial to do so (they were given 4 second to make a choice but did so on average within 1.91 s, ±0.26).

Examination of the choice times also revealed that participants were substantially faster in choosing between colors than between $\text{Cs}\left(t_{87}=19.45,p<0.001\right)$. This raised the possibility that the tradeoff that was evident in the combined trials was the result of fast gathering of learned information, leading to lesser need to use perceived information to reach a decision threshold. If this is the case, we may expect to see that participants fixated on fewer Cs in combined trials, compared to the Cs-only trials. The data showed this was indeed the case ($t_{71}=8.7,p<.001$; Figure 2D). Moreover, both this effect and the shorter choice time in combined trials correlated with the degree to which participants prioritized colors over Cs in making their choices, as inferred from participants’ choices ($\Sigma {\text{C}}_{\text{observed}}:\text{r}=-0.4,t_{70}=3.66,p<.001$; Figure 2F; choice time: r = −.39, $t_{86}=-4,p<.001$, Figure 2G), and validated by participants’ self-reports concerning their use of Cs and colors (r = 0.55, p<.001, Figure 2E).

The finding that participants were faster to choose when stimuli also involved color put into question the assumption that gathering information about the color takes substantial time, similarly to the gathering of the perceptual information. To explicitly test this, we added to our logistic regression model of choice an interaction between the color predictor and choice time, while still controlling for the association of color use with fewer observed Cs. The results did not provide evidence that using the colors took additional time. In fact, the interaction of color use and choice time was negative $\left(\beta_{\text{RTXcolor}}=-0.09,z=-2.6,p=0.009\right)$. Possibly, this result reflects that the better the color reward probabilities were learned, the faster it was to use them.

### A computational model of perceptual and learned information gathering

The results thus far establish a trade-off between the use of learned and perceived information, and suggest that this trade-off results because fast, initial gathering of learned evidence gets people closer to a decision threshold, and thus reduces the amount of perceptual information they need to gather in order to reach a decision. To formally test this mechanism, we leveraged the traceability and discreteness of perceptual information gathering in our task to form a computational model of the task. Thus, the model predicted not only the choices participants made, but also the sequence of fixations that would lead them to each choice (see Methods for full model specification).

At each timepoint, the model first decides whether to gather more perceptual information or already make a choice. This decision can lead to a tradeoff between perceptual and learned information because, among other factors, it is based on the absolute difference in the currently estimated values of the stimuli’s colors $\left(\Delta {\text{V}}_{\text{color}}\right)$ :

(1) $$\text{p}(\text{continue}\;\text{gathering})=\text{logistic}\left(\alpha_{\text{gather}}+\beta_{\Delta \text{Cs}}\left|\Delta {\text{V}}_{\text{Cs}}\right|+\beta_{\Delta \text{color}}\left|\Delta {\text{V}}_{\text{color}}\right|+\right.\left.\beta_{\text{time}}\left(1-\tau^{\text{trial-t}}\right)\right)$$

Specifically, a tradeoff would result if $\beta_{\Delta \text{color}}$ is negative. Other factors influencing this decision are the value difference according to the observed $\text{Cs}\left(\Delta {\text{V}}_{Cs}\right)$, the model’s general tendency to gather or not gather perceptual information ($\alpha_{\text{gather}}$), and its urgency to make a decision as time goes by from trial onset (trial-t). Here, τ∈[0,1] determines how quickly urgency grows with time.

If the model decides to gather additional perceptual information, it then chooses whether to look at the right or left stimulus’ Cs:

(2) $$\text{p}(\text{look}\;\text{right})=\text{logistic}\left(\alpha_{\text{look-right}}+\beta_{\text{look-color}}\Delta {\text{V}}_{\text{color}}+\beta_{\text{look-Cs}}\Delta {\text{V}}_{\text{Cs}}+\right.\beta_{\text{look-stay}}I(\text{looked}\;\text{right})$$

This choice is based on a general bias to look in one or another direction ($\alpha_{\text{look-right}}$), the values of the right versus left stimuli, and where the model last looked at (since it is less effortful to saccade within a stimulus). Here, I (looked right) is coded as +1 if the model last looked right, and −1 if the model last looked left.

When the model decides to stop gathering perceptual information, it chooses either the right or the left stimulus, based on their estimated values:

(3) $$\text{p}(\text{choose}\;\text{right})=\text{logistic}\left(\beta_{\text{choose}}\Delta V\right)$$

where $\beta_{\text{choose}}$ is an inverse temperature parameter, and ΔV accounts for both colors and Cs:

(4) $$\Delta V=\Delta {\text{V}}_{Cs}+\omega_{\text{color}}\Delta {\text{V}}_{\text{color}}$$

where $\omega_{\text{color}}$ weights the relative impact of the colors on choice.

Lastly, the value of a stimulus based on its observed Cs is computed as a mean of the expected reward for 3, 6, or 9 upward-facing Cs, each multiplied by the likelihood that there were this number of upward-facing Cs given the observed Cs (as per the binomial probability mass function). And the value added due to the stimulus having a particular color is learned on a trial-by-trial basis from the observed rewards (see methods). To account for the fact that retrieving the learned color values could take time, for instance due to a process of sampling from stored memory^14^, the effective value used for making choices increasingly approaches the learned value as time within a trial progresses:

(5) $${\text{V}}_{\text{color}}={\text{V}}_{\text{color}}^{\text{learned}}\left(1-\tau_{\text{color}}^{\text{trial-t}}\right)$$

with the rate of increase determined by $\tau_{\text{color}}$.

### Learned values impact perceptual information gathering

**Figure 3.**
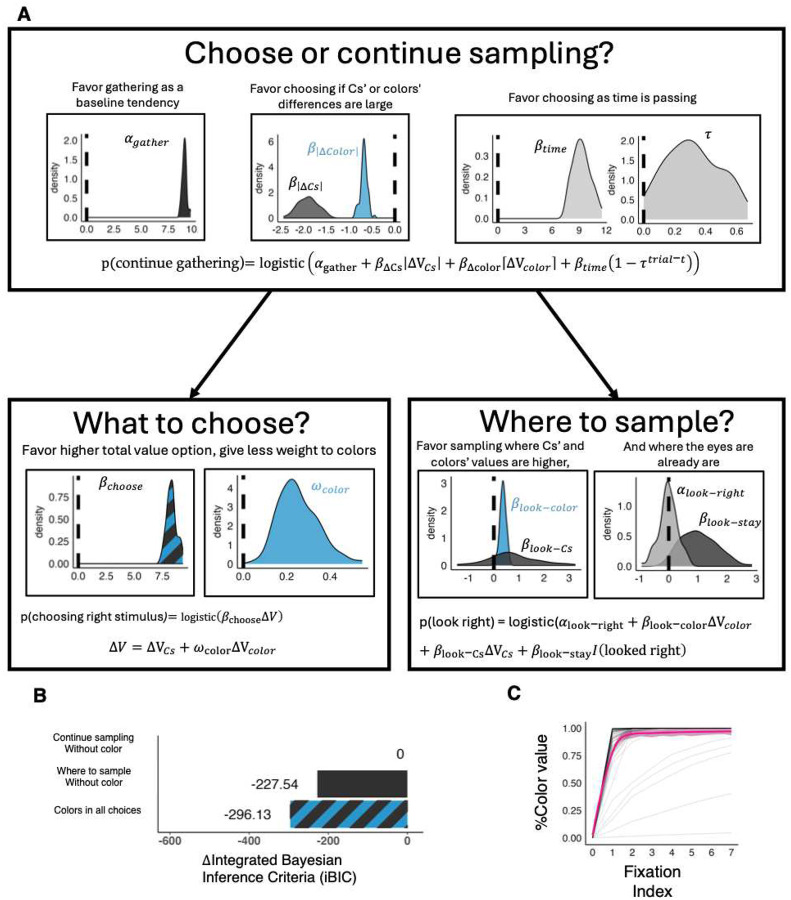
Modelling results. A. The plots show the fitted distributions of group-level mean parameters. Top: Parameters governing the decision of whether to choose a stimulus or to look for more information. The general tendency of participants is to look for more information (left plot). This decision, however, also depends on the absolute difference between the currently estimated values of both colors and Cs. The higher the differences, the more participants are likely to stop gathering information and choose (middle plot). Participants were also less likely to continue gathering perceptual information as time passed (right plot). Bottom: Parameters governing the decision of where to look and what to choose. In choosing a stimulus, participants favored stimuli with higher total value, but gave only partial weight to the color in computing this value (left plots). The decision of where to look was consistently governed by a perseverance factor ($\beta_{\text{look-stay}}$) and by the value associated with the stimuli’s color (right plots). B. Comparison of integrated Bayesian inference criteria between models excluding color information either from the decision of whether to sample more information or to choose, or from the decision of where to sample from, showing that the best fitting model is the one that incorporates colors in both. C. Color value gathered as a function of fixation number, showing the majority of participants gathered over 90% of the color value within 2 fixations. Black lines depict individuals; magenta line is group average.

Fitting the model to participants’ actual gaze choices (fixations) and choices showed that choices of whether to continue gathering information, and choices of where to sample from, were influenced by both Cs and color information. Thus, the best-fitting model according to integrated Bayesian Inference Criteria^17,18^ (iBIC = 182139.3) incorporated learned color information in both the decision of whether to continue gathering information and the decision of where to sample. This full model outperformed models that included learned color information only in the gather-versus-choose decision (iBIC = 182435.46) or only in the sampling location decision (iBIC = 182207.92; Figure 3B).

Examining the parameter values that best fitted participants’ choices and gaze fixations showed the decision to continue gathering perceptual information was negatively influenced by the color values – the higher the absolute difference between the color values, the lower the probability of continuing to gather perceptual information. Moreover, color values were gathered very rapidly by the model, on average reaching 94.6% of the learned color value already in the second fixation (see Figure 3C). Thus, mirroring the model-independent results, the modelling indicated that the tradeoff between learned and perceived information resulted from a fast retrieval of learned information that suppressed further gathering of perceptual information.

**Figure 4.**
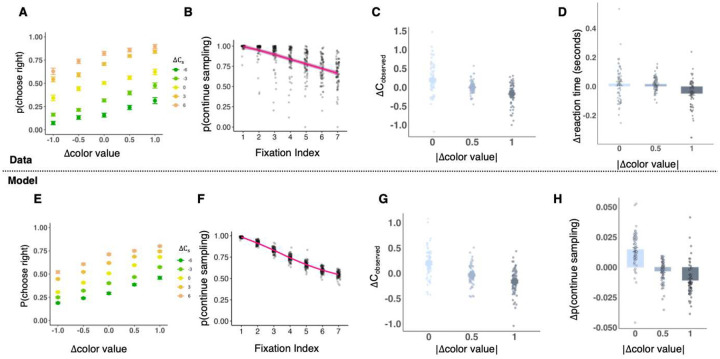
Correspondence between modeling results and data. A. Participants’ probability of choosing the right stimulus as a function of the difference in the reward probability associated with the stimuli’s colors, and the difference in number of Cs pointing upwards (indicated by the colors). Error bars indicate participants-level standard error. B. The probability of looking at another C given the number of previous fixations on Cs. Each dot represents a participant. Line represents group average. C. Participants looked at fewer Cs when the absolute difference between stimulus color values was larger. Total Cs observed are centered within participants; each dot represents a participant. D. Participants chose faster when the absolute difference between stimulus color values was larger. RT are centered within participants; each dot represents a participant. E. The model’s probability of choosing the right stimulus as a function of the difference in color value (indicated by the X axis), and in the value of the observed Cs (indicated by the colors), between the stimuli. Each dot is a decision (choosing right or left) made in the experiment. F. The model’s probability of choosing to continue sampling as a function of fixation index. G. The number of Cs observed by the model was lower when the absolute difference between the color values was larger. H. The model’s probability of continuing to sample changed as a function of the absolute difference between the color values.

To confirm that the model captured participants’ behavior well, we compared it to the actual data in terms of both stimulus choices and decisions to continue looking. With regards to stimulus choices, we examined the probability of choosing the right or left stimulus as function of the reward probabilities associated with the stimuli’s color and Cs. This showed an adequate correspondence between participants (Figure 4A) and model (Figure 4E). With regards to decisions to continue looking, we examined the probability of gathering additional perceptual information following each additional fixation. This measure too showed a good correspondence between participants (Figure 4B; β=-0.06, t = 29.4, p < .001, mixed regression) and model (Figure 4F; β=-0.07, t = 148.8, p < .001, mixed regression). Finally, we examined measures that stem from the mechanism underlying the tradeoff between learned and perceived information: the reduced tendency to continue looking at more Cs the higher the absolute differences between the color values. This reduced tendency was evident in the number of Cs observed (Figure 4C; β=-0.36, t = 7.28, p < .001, mixed regression) and the time it took participants to stop looking and choose a stimulus (Figure 4D; β=-0.07, t = 3.66, p < .001, mixed regression). These measures aligned well with the number of Cs observed by the model (Figure 4G; β=-0.009, t = 6.5, p < .001, mixed model) and the model’s probability of continuing to look (Figure 4H; β=-0.018, t = 12.01, p < .001, mixed regression).

### Individual differences and reliability in the use of learned and perceived information

The results so far established and explained a trade-off between learned and perceived information within participants. We finally asked whether a trade-off also exists *between* individuals, such that the more a person relies on one source of information, the less they rely on the other. To test this, we examined the correlation between the regression coefficients quantifying the degree to which each participants used the Cs and the colors to make choices. This planned analysis showed a weak, non-significant negative correlation (r = −0.16, p = .14; Figure S1A), suggesting that our measures might not be sensitive enough given the present sample size.

We thus repeated the correlation analysis replacing the regression coefficients with the parameter values for the weights participants gave to continue gathering $\text{Cs}(\alpha_{\text{gather}})$ and color ($\omega_{\text{color}}$) as derived from the modelling of participants’ choices and fixations. The modelling more specifically quantifies participants’ baseline tendency to gather Cs and their usage of color information to which they were exposed. We found a negative correlation across participants between the two parameters, such that the more participants used the color information the less they gathered information about the Cs and vice versa (r = −0.23, p=0.04; Figure S1B). We also note that this result likely underestimates the true magnitude of the trade-off because it may reflect a superposition of two correlations, one negative due to a trade-off and another positive due to individual differences in general performance, for instance due to motivation, which would lead to higher or lower values in both coefficients.

**Figure 5.**
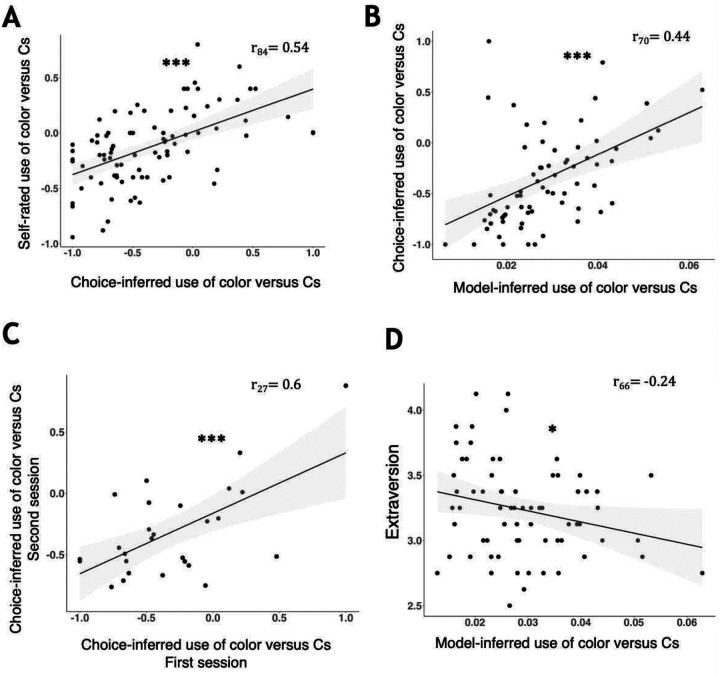
Individual differences in the use of perceived versus learned information. *: p<.05. ***: p<.001. Each point represents a single participant. A. The relationship between the self-reported ratings of the degree to which participants relied on color vs Cs in making choices, to the ratio of choices-derived regression coefficients ($\frac{\beta_{\text{color}}-\beta_{\text{Cs}}}{\left(\left|\beta_{\text{Cs}}\right|+\left|\beta_{\text{color}}\right|\right)}$; see Figure 2). B. The relationship between choices-derived regression coefficients and the ratio of information gathering/usage parameters from the model $\left(\frac{\omega_{\text{color}}}{\alpha_{\text{gather}}}\right)$. The number of participants is lower here because modeling required valid eye tracking data not available for all participants. C. Reliability of information gathering style in the task, quantified as in panel A, across two sessions of the experiment performed at least 2 days apart. D. Trait Extraversion as a function of information gathering style in the task, quantified using the model as in panel B. The number of participants is lower here because not all participants filled the questionnaire.

In addition, we wanted to examine whether participants’ tendency to prioritize perceived or learned information in making choices is a stable and valid individual trait. For this purpose, we first examined the correlation between the color-Cs relative coefficient measure (see Figure 2C) in two separate sessions of the experiment, each with different colors, that participants completed in two different days (between 2 and 175 days apart, mean 79 ± 63). A positive correlation (n = 27, r = 0.6, p<.001) indicated moderate test-retest reliability suggesting some degree of stability in individuals’ tendency to use perceived versus learned information in our task (Figure 5C).

To test the validity of these individual differences, we next examined whether they correlated with participants’ own ratings of how much they used the colors and the Cs. This showed a significant positive relationship (Figure 5A), indicating a correspondence between the task measure and participants’ self-reports. We next tested the validity of the task measure for explaining behavior outside the task, specifically, by assessing its correspondence with personality self-reports (Big 5^19^). We hypothesized that greater use of perceived, relative to learned, information would be associated with trait extraversion and openness to experience. We found a trend-level correlation with extraversion (r = .21, p = 0.057) but not with openness to experience (.15, p = 0.33; all correlations appear in Table S1). We thus repeated the analysis with respect to extraversion using the more specific task measures derived from the modeling, which were moderately correlated with the measures derived from a regression analysis of choices (Figure 5B). This showed a significant correlation (r = −.24, p = 0.04, Figure 5D), such that more extraverted participants gathered more perceptual information relative to their usage of learned information.

## Discussion

Using a novel pre-registered experiment that required participants to jointly use learned and perceived information to form decisions, we showed that there is a trade-off between the use of learned information and the gathering of new, perceptual information, both within- and between-participants.

The data indicated that learned information is fast to gather reducing the need for the effortful gathering of additional perceptual information. Moreover, learned information directs the gathering of perceptual information towards higher-value options^15,20–22,22–26^, and thus leads one to gather perceptual information that is more likely to generate a decision.

This work illustrates how humans dynamically integrate internal and external sources of information to flexibly adapt their exploratory strategies. Crucially, it shows how humans decide not only whether to seek additional information, but also *which* information to pursue. These findings are particularly relevant for understanding ecological human behavior, where information seeking is not an isolated event but a continuous, multidimensional process that underpins adaptive action in complex environments^23,27–39^.

Prior work has primarily considered similarities between the gathering of externally perceived and internally stored information^27,40–45^, suggesting that attention gathers information from our senses, while working memory does so from within the mind^27^. Indeed, comparable patch-like information structures and search behaviors were found in both domains. Thus, principles drawn from research about foraging^43,46^ have been used to explain how we retrieve semantic information from memory^47–49^, do creative search^50^, or shift our gaze to areas rich in semantic or visually dense information^26^. Here we extend prior work by showing how the two sources of information compete in influencing decision making.

Importantly, our findings emerged within an experimental design in which perceptual information could only be accessed through eye movements. This requirement reflects a naturalistic feature of decision-making, but the precise time cost of eye movements is likely to vary across contexts, raising the possibility that the strength of the trade-off we observed may itself adapt to environmental demands^51^.

In addition to the trade-off within individuals, participants’ overall tendency to prioritize one source of information over the other emerged as a stable individual trait across two separate sessions of the experiment. This characteristic was reflected in the modeling results and had a marginally significant correlation with trait extraversion. These results suggest a stable latent individual preference for relying on either internally gathered or externally sampled information, with the latter motivating extraverted behavior. Indeed, trait extraversion has been associated with a greater sensitivity to external stimuli and a preference for externally sourced information^52^. Here, we characterize how this personality tendency may manifest in learning and decision-making. Given our marginally significant result, further research employing larger samples is necessary to establish our conclusions regarding trait differences.

Future work could also examine relationships with additional traits that potentially originate from a preference for gathering perceptual information. One such trait is emotional temperament, in particular, proneness to depression which is known to be associated with a tendency to ruminate internally while avoiding activities that can expose one to new external information^53^.

Another promising avenue for further research concerns the information provided by each source. In our experiment, learned and perceived information provided the same amount and kind of information – probability of reward. This feature of our task can be manipulated, for instance, by having one source of information convey reward probability and the other reward magnitude, which could potentially serve to reduce the tradeoff between them.

In conclusion, our findings extend current understanding of how humans combine newly perceived and previously learned information^54^ by establishing a trade-off between these two sources of information in guiding decisions, highlighting that beneath each choice lies a more fundamental decision about which source of information to consult.

## Methods

### Participants

The sample included 90 university students with normal or corrected-normal vision (62 women; 66 with right dominant eye; average age 23.5, sd 3.03), all scoring 70 or above in the Ishihara color blindness test. We based our sample size on a power analysis as pre-registered in https://osf.io/78vqu and detailed below. Two participants were removed from behavioral data analyses because their performance in combined color-Cs trials, measured as the proportion of choices of stimuli associated with the higher probability of reward, was not higher than chance level (50%). Valid eye tracking data (above 75% valid samples) were obtained from 72 participants (see exclusion criteria under Eye tracking) and inserted into the eye movements analysis. To test the reliability of task measures, 29 participants were invited back for another session of the experiment, out of which 27 met the performance criterion. All participants signed an informed consent before the experiment, approved by the Ethics review board of the Hebrew University of Jerusalem. They were granted either course credit or 40 NIS (~10$), in addition to 0.3NIS (~0.01$) for each reward obtained in the experiment. Due to dropout and participants returning rates, only 51 filled the ATQ questionnaire, and 68 filled the big5 questionnaire.

### Power analysis

We performed a power analysis to determine the required sample size for the across-participants trade-off analyses, as such analyses require larger sample sizes. We expected a medium effect size, which entails a correlation of approximately 0.3, and required a power of 0.8 at a significance level of p=0.05. This necessitated a sample size of at least 84 participants. For testing whether the prioritization of internal or external information is a reliable disposition, we expected a large effect size of at least r=0.5 in a test-retest correlation across participants. For this analysis, a power of 0.8 at significance of p=0.05 necessitated a sample size of at least 28 participants.

### Procedure

Participants were first familiarized with the experimental stimuli and instructed that reward probability scales with the number of upward-facing Cs, which could be either 3, 6, or 9. Additionally, participants were told that the reward probability associated with the colors can be learned by trial and error. To maximize their reward, participants were instructed to give weight to both Cs and colors in choosing between combined stimuli, because the contributions of the two components of the stimuli to predicting reward are additive.

The experiment started with at least 10 practice trials of each type – Cs only, color only, and their combination - each of which continued until the participant reached at least 80% accuracy:

Then, participants played three blocks of trial. The first and second block each introduced three new colors, associated with different reward probabilities (0, 0.5, and 1). The colors associated with each probability were counter-balanced across participants. To ensure participants learned about the colors and understood the Cs trials properly, 36 combined stimuli trials were interleaved with 18 Cs-only trials and 18 color-only trials. In the third block, participants faced all six colors they had already learned about. Since no new colors were introduced in this phase, this block consisted only of 162 combined trials.

Following every 30 trials, participants rated how they felt on valence and arousal on visual analog scales. At the end of the experiment, participants reported the reward probability associated with each color and rated the degree to which they relied on the Cs versus the color in making their choices on a visual-analog scales, used to calculate the ratings in Figure 7A. Finally, participants were requested to fill out a curiosity and personality questionnaire – big5 and ATQ^19,55^.

### Eye tracking

The experiment began with a standard 9-point calibration and validation procedure provided by Eyelink 1000+ (SR Research Ltd., Mississauga, Ontario, Canada). The eye-tracking measures are based on EyeLink’s standard parser configuration: samples were defined as a saccade when the deviation of consecutive samples exceeded 30 °/s velocity or 8,000 °/s^2^ acceleration. Samples gathered from time intervals between saccades were defined as fixations.

The eye tracking data was parsed into fixation reports, and interest areas were defined around each of the Cs. We discarded repeat fixations (fixations on a C that has already been fixated on in the same trial) from further analysis, assuming that if another fixation was made to the same location it means that the subjects did not register its orientation the first time. We then calculated for each participant how many of each stimulus’ Cs the participant has already fixated on at each time point, and how many of these faced upwards or downwards.

### Regression models

Several mixed regression models were run using the lme4 package in R. To validate that participants use both Cs and color, we compared three regression analyses explaining participants’ choices.

1. the difference between the two available stimuli in the number of upward Cs(Cs):
Choice~Cs+(1+Cs∣subject)
2. the difference between the stimuli in the reward probability associated with the color (*color*):
Choice~color+(1+color∣subject)
3. both:
Choice~Cs+color+(1+color+Cs∣subject)

To assess the hypothesized trade-off between the use of Cs and color to make choices, we examined the interaction between the use of color and the overall number of Cs participants fixated on:

$$\text{Choice}\sim \Delta {\text{Cs}}_{\text{observed}}+\Delta \text{p}(\text{reward}|\text{color})+\Delta \text{p}(\text{reward}|\text{color})\cdot \Sigma {\text{Cs}}_{\text{observed}}+(1+\Delta {\text{Cs}}_{\text{observed}}+\Delta \text{p}(\text{reward}|\text{color})+\Delta \text{p}(\text{reward}|\text{color})\cdot \Sigma {\text{Cs}}_{\text{observed}}|\text{subject})$$

where $\Delta {\text{Cs}}_{\text{observed}}$ encodes how many upwards facing minus downwards facing Cs participants fixated on during the trial for the right minus left stimulus, $\Sigma {\text{Cs}}_{\text{observed}}$ is the overall number of Cs the participant fixated on, and Δp(reward|color) is the difference between the stimuli in the reward history of their colors, that is, the proportion of times that choosing a stimulus with the color was rewarded.

Then, to test whether retrieving the reward history of a color required time, we added the reaction time (RT) as another moderator of the color predictor:

$$\text{Choice}\sim \Delta {\text{Cs}}_{\text{observed}}+\Delta \text{p}\left(\text{reward}|\text{color}\right)+\Delta \text{p}\left(\text{reward}|\text{color}\right)\cdot \Sigma {\text{Cs}}_{\text{observed}}+\Delta \text{p}\left(\text{reward}|\text{color}\right)\cdot \text{RT}+(1+\Delta {\text{C}}_{\text{observed}}+\Delta \text{p}(\text{reward}|\text{color})+\Delta \text{p}(\text{reward}|\text{color})\cdot \Sigma {\text{Cs}}_{\text{observed}}+\Delta \text{p}\left(\text{reward}|\text{color}\right)\cdot \text{RT}|\text{subject})$$

All predictors were normalized within participants.

### Modeling

The computational models were fit to the data using a custom iterative importance sampling algorithm, implemented in python. This allowed us to extract max-likelihood estimates both for the hyper parameters and for participant-level parameters. The major part of the model is described in the main text. Here we complement the description by specifying additional components.

#### Value of color ($V_{\text{color}}$)

The value associated with each color was updated by a temporal difference learning algorithm:

(s1) $$V_{{\text{chosen-color}}_{t}}^{\text{learned}}\leftarrow V_{{\text{chosen-color}}_{t}}^{\text{learned}}+\eta_{t}\cdot \delta$$

where the prediction error, δ, is computed as:

(s2) $$\delta =r-\left(V_{{\text{chosen-Cs}}_{t}}^{\text{learned}}+V_{{\text{chosen-color}}_{t}}\right)$$

and the learning rate $\eta_{t}$ adapts based on the number of prior observations for each color stimuli:

(s3) $$\eta_{t}=\frac{1}{\epsilon +\frac{1}{2}n_{{\text{chosen-color}}_{t}}}$$

Here ϵ is a free parameter, and the number of observed outcomes is divided by 2 because each outcome is only half attributable to the color.

#### Value of C ($V_{\text{CS}}$)

The value of each side’s Cs was computed as the likelihood of observing the number of upward-facing Cs, using a binomial probability density function based on all Cs observed on that side. The likelihood for a given number of upward-facing Cs (n) out of the total observed Cs is calculated as:

(s4) $$p\text{(Cs-up}=n|\text{observed}\;\text{Cs})\propto {\left(\frac{n}{12}\right)}^{\text{observed}\;\text{up}}{\left(1-\frac{n}{12}\right)}^{\text{observed}\;\text{down}}$$

where n/12 represents the probability of observing an upward-facing CS, and the exponents represent the actual counts of upward and downward-facing Cs observed. The overall value for each side is then computed as a weighted sum across possible numbers of upward-facing CSs:

(s5) $$V_{s\text{-Cs}}=\text{p}(s\text{-Cs-up}=3)0.75+\text{p}(s\text{-Cs-up}=6)0.5+\text{p}(s\text{-Cs-up}=3)0.25$$

#### Cs only trials

For Cs-only trials, color predictors were set to zero, and the expected value of a stimulus was computed as:

(s5) $${\text{V}}_{\text{Cs}}=p(9)*1+p(6)*0.5+p(3)*0$$

#### Color only trials

For color-only trials, Cs predictors were set to zero. Thus, choice probabilities were modelled as a logistic function of $\beta_{\text{choose}}\omega_{\text{color}}\left(V_{\text{right}}-V_{\text{left}}\right)$.

#### Hyper priors

All β and α parameters were each drawn for each participant from a separate group-level normal distribution, with a mean of 0 and a standard deviation of 1. The prior distribution for the $\omega_{\text{color}},\tau$ and $\tau_{\text{color}}$ parameters were specified as approximately uniform distribution ranging [0, 1]. To achieve this normally distributed values (μ=0,σ=1) were transformed using the cumulative distribution function (CDF) of the normal distribution. This approach ensured that parameter values remained constrained to [0, 1], while allowing the distribution to adapt during the fitting process. For the parameter ϵ, we specified a lognormal prior with mean 2 and standard deviation 2, chosen as a weakly informative, wide prior.

## Data Availability

Data will be available upon request from Tal Nahari.

## Code availability

Code will be available upon request from Tal Nahari.
